# Non-image Forming Light Detection by Melanopsin, Rhodopsin, and Long-Middlewave (L/W) Cone Opsin in the Subterranean Blind Mole Rat, *Spalax Ehrenbergi*: Immunohistochemical Characterization, Distribution, and Connectivity

**DOI:** 10.3389/fnana.2016.00061

**Published:** 2016-06-09

**Authors:** Gema Esquiva, Aaron Avivi, Jens Hannibal

**Affiliations:** ^1^Department of Clinical Biochemistry, Bispebjerg Hospital, University of CopenhagenCopenhagen, Denmark; ^2^Department of Physiology, Genetics and Microbiology, University of AlicanteAlicante, Spain; ^3^Laboratory of Biology of Subterranean Mammals, Institute of Evolution, University of HaifaHaifa, Israel

**Keywords:** rhodopsin, L/M cone opsins, amacrine cells, bipolar cells, Brn3a, calretinin, RBPMS, Ctbp2

## Abstract

The blind mole rat, *Spalax ehrenbergi*, can, despite severely degenerated eyes covered by fur, entrain to the daily light/dark cycle and adapt to seasonal changes due to an intact circadian timing system. The present study demonstrates that the *Spalax* retina contains a photoreceptor layer, an outer nuclear layer (ONL), an outer plexiform layer (OPL), an inner nuclear layer (INL), an inner plexiform layer (IPL), and a ganglion cell layer (GCL). By immunohistochemistry, the number of melanopsin (mRGCs) and non-melanopsin bearing retinal ganglion cells was analyzed in detail. Using the ganglion cell marker RNA-binding protein with multiple splicing (RBPMS) it was shown that the *Spalax* eye contains 890 ± 62 RGCs. Of these, 87% (752 ± 40) contain melanopsin (cell density 788 melanopsin RGCs/mm^2^). The remaining RGCs were shown to co-store Brn3a and calretinin. The melanopsin cells were located mainly in the GCL with projections forming two dendritic plexuses located in the inner part of the IPL and in the OPL. Few melanopsin dendrites were also found in the ONL. The *Spalax* retina is rich in rhodopsin and long/middle wave (L/M) cone opsin bearing photoreceptor cells. By using Ctbp2 as a marker for ribbon synapses, both rods and L/M cone ribbons containing pedicles in the OPL were found in close apposition with melanopsin dendrites in the outer plexus suggesting direct synaptic contact. A subset of cone bipolar cells and all photoreceptor cells contain recoverin while a subset of bipolar and amacrine cells contain calretinin. The calretinin expressing amacrine cells seemed to form synaptic contacts with rhodopsin containing photoreceptor cells in the OPL and contacts with melanopsin cell bodies and dendrites in the IPL. The study demonstrates the complex retinal circuitry used by the *Spalax* to detect light, and provides evidence for both melanopsin and non-melanopsin projecting pathways to the brain.

## Introduction

The mole rat *Spalax ehrenbergi* (muroid family Spalacidae), is a blind subterranean mammal with rudimentary eyes located under the skin. While completely blind (Cernuda-Cernuda et al., 2002), the *Spalax* responds to light stimulation and is able to adapt behavior and physiology to the 24 h solar cycle as well as seasonal changes (David-Gray et al., 1998; Nevo et al., 2001). Despite that embryonic development appears normal, the adult eye of the *Spalax* has a degenerate anterior chamber, iris-ciliary complex and lens, while the retina retains its morphologic integrity, with well-organized layers, but less organized than in sighted mammals (Cernuda-Cernuda et al., 2002). The eyes are < 1 mm in diameter and the regressed optic nerve contains < 900 axons. Functional studies have confirmed that *Spalax* has no image-forming vision (Cooper et al., 1993a), and it has been suggested the *Spalax* eye functions as a light meter corresponding to the non-images forming system (NIF) found in the sighted eye (Cooper et al., 1993b; Hannibal et al., 2002b). This notion is supported by retinal tract tracing showing that brain involved primarily in visual perception receives markedly reduced retinal projections while areas involved in NIF functions (circadian timing) such as the suprachiasmatic nucleus (SCN) and the ventral geniculate nucleus (VGL) are innervated similarly to that of sighted animals (Bronchti et al., 1991; Cooper et al., 1993b). Within the last decade understanding of the NIF system of the sighted eye has been markedly increased by anatomical and functional observations. The major discovery was the identification of the photopigment melanopsin found in a subset of intrinsically photosensitive retinal ganglion cells (ipRGCs) (Hattar et al., 2002; Hannibal et al., 2002a). Initial studies showed that light via the ipRGCs entrained circadian rhythm independent of the rods and cones (Hattar et al., 2003). It became clear, however, that melanopsin expressing RGCs (mRGCs) in addition with input from rods and cones regulated circadian timing. Furthermore, several subtypes of mRGCs (in mice M1-M5), wired from rods and cones contributed to light entrainment of the circadian system (Hattar et al., 2003; Lucas et al., 2003; Schmidt and Kofuji, 2010; Schmidt et al., 2011a). These observations indicated that the non-image forming system in sighted eyes was more complex than previously suggested (Schmidt et al., 2011a; Jagannath et al., 2015). In light of this, it therefore became interesting to re-examine the *Spalax* retina, which despite melanopsin (Hannibal et al., 2002b) has been shown to express a functional rhodopsin and a long/middle wave (L/M) cone opsin (Janssen et al., 2000, 2003). A more detailed anatomical understanding of the retina of the *Spalax* can provide information of the complexity of the NIF system in this animal, and in addition be used to understand the NIF system of the sighted eye.

The present study provides, using immunohistochemistry and retinal markers for mRGCs, rods, cones, amacrine, and bipolar cells in combination with synaptic markers, a detailed investigation of melanopsin bearing retinal ganglion cells and their synaptic contacts with other retinal cells.

## Materials and methods

### Animals

Six male blind mole rats, *Spalax Judaei*, belonging to the superspecies *Spalax Ehrenbergi* (Nevo et al., 2001) from the Anza population in Samaria, kept in a 12:12 h light/dark cycle were used for the study. All animals were anesthetized with tribromoethanol (250 mg/kg, i.p.) and transcardially perfused with Stefanini's fixative (2% paraformaldehyde, 0.2% picric acid in 0.1 M sodium phosphate buffer, ph 7.2). The eyes, located in the harderian gland, were removed, postfixed overnight in the same fixative solution, cryoprotected in 30% sucrose and stored at –20°C until immunohistochemically processed. Experiments were performed according to the Ethical principles of Laboratory Animal Care (Law on Animal Experiments in Denmark, publication 1306, November 23, 2007) and Dyreforsoegstilsynet, Ministry of Justice, Denmark. All animals were killed between Zeitgeber (ZT) 4-8 (ZT0 = lights on).

### Antibodies and immunohistochemistry

All antibodies used in the study are listed in Tables 1, 2. A C-terminal rabbit polyclonal anti-melanopsin antibody (code no.41k9, diluted 1:5000) characterized previously (Hannibal et al., 2002a) was used for all melanopsin staining.

**Table 1:** **Primary antibodies**.

**Molecular marker**	**Antibody**	**Source**	**Species**	**Working dilution**
Brn3a	Sc31984	Santa Cruz Biotechnology Cat # sc-31984 RRID:AB_2167511	Goat	1:500
Calretinin	7699/4 Lot:18299	Swant Cat# 7699/4 RRID:AB_2313763	Rabbit	1:500
ChAT	144P	Millipore Cat# AB144P-1ML RRID:AB_262156	Goat	1:500
Ctbp2	612044	BD Biosciences Cat# 612044 RRID:AB_399431	Mouse	1:2K
Melanopsin	41K9	In house	Rabbit	1:1K-10K
L/M coneopsin	JH492	J. Nathans, Johns Hopkins University School of Medicine; Maryland; USA Cat# JH 492 RRID:AB_2315259	Rabbit	1:5 K
PKC	Sc-12356	Santa Cruz Biotechnology Cat# sc-12356 RRID:AB_2168557	Goat	1:500
PKC	P-5704 (CloneMC5)	Sigma-Aldrich Cat# P5704 RRID:AB_477375	Mouse	1:500
PKC	P4334	Sigma-Aldrich Cat# P4334 RRID:AB_477345	Rabbit	1:5000
PACAP	MabJHH1	J. Hannibal, Bispebjerg Hospital, University of Copenhagen; Copenhagen; Denmark Cat# Mab JHH1 RRID:AB_2315043	Mouse	1:5
Recoverin	K21+	Karl-W Koch	Rabbit	1:2000
RBPMS	1832	PhosphoSolutions Cat# 1830-RBPMS RRID:AB_2492225	Gineapig	1:500
Rhodopsin	Rho-4D2	David Hicks, R. Molday, University of British Columbia; British Columbia; Canada Cat# rho4D2 RRID:AB_2315273	Mouse	1:1K
S-cone opsin	JH455	J. Nathans, Johns Hopkins University School of Medicine; Maryland; USA Cat# short-wavelength-selective cone opsin (JH455) RRID:AB_2315324	Rabbit	1:5K
	Sc-14363	Santa Cruz Biotechnology Cat# sc-14363 RRID:AB_2158332	Goat	1:200
TH	MAB318	Millipore Cat# MAB318 RRID:AB_10050306	Mouse	1:1K
TH	AB151	Millipore Cat# AB151 RRID:AB_10000323	Rabbit	1:1K
VIP	291E	Fahrenkrug et al., 1995 Cat# 291E RRID:AB_2313759	Rabbit	1:2K-1:10K

**Table 2:** **Secondary antibodies**.

**Antibody**	**Source**	**Species**	**Working dilution**
Biotinylated donkey antiserum	Jackson ImmunoResearch Labs Cat#711-065-152 RRID:AB_2340593	Rabbit	1:800
Biotinylated donkey antiserum	Jackson ImmunoResearch Labs Cat# 715-065-151 RRID:AB_2340785	Mouse	1:800
AlexaFluor 594-conjugated donkey IgG	Thermo Fisher Scientific Cat# A21207 RRID:AB_10049744	Rabbit	1:800
AlexaFluor 594-conjugated donkey IgG	Jackson ImmunoResearch Labs Cat# 706-585-148 RRID:AB_2340474	Ginea Pig	1:200
AlexaFluor 488-conjugated donkey IgG	Jackson ImmunoResearch Labs Cat# 705-096-147 RRID:AB_2340402	Goat	1:800
AlexaFluor 488-conjugated donkey IgG	Jackson ImmunoResearch Labs Cat# 715-545-151 RRID:AB_2341099	Mouse	1:200
Dylight (Cy5)conjugated donkey IgG	Jackson Immunoresearch 705-496-147	Goat	1:200
Cy5-conjugated donkey	Jackson Immunoresearch 715-055-150	Mouse	1:200
Bioinylated tyramide	DuPon NEN700		1:1
Streptavidin-conjugated AlexaFluor 488			1:500
Envision	Dako (K4002)	Rabbit	1:2
Envision	Dako (K4000)	Mouse	1:2
Tyramide-conjugated Alexa594	Molecular Probes		1:300
Tyramide-conjugated Alexa488	Molecular Probes		1:300

Anti-melanopsin antibody was used in combination with a series of other antibodies (Table 1). To identify retinal cell bodies, we mounted sections in glycerol/water added with the DNA-binding AT-specific fluorochrome 4′-6-diamino-2-phenylindole (DAPI).

Secondary antibodies used are present in Table 2. In cases where two primary antibodies were raised in the same species we used a combination of biotinylated tyramide (tyramide system amplification; DuPon NEN, Boston, MA), and streptavidin-conjugated AlexaFluor dyes or Envision (Dako, ChemMate, Glostrup, Denmark) and tyramide-conjugated Alexa dyes (Table 2).

For retinal sections, eyeballs embedded in Tissue-Tek® O.C.T. (Sakura Finetek INC, USA) were cut in 12 μm thick sections in series of three in a cryostat. The sections were mounted on Superfrost slides, dried and frozen at –20°C until processed for immunohistochemistry (IHC). Some sections were pretreated with antigen retrieval solution for 24 h at 40°C (Dako ChemMate, Glostrup, Denmark; code No. S 203120 in distilled water, pH 6) before processing for IHC. A single flat-mount retina was prepared from one animal by removing the cornea and lens and the retina was dissected out from the choroid and treated with antigen retrieval solution for 1.5 h at 80°C (Dako ChemMate, Glostrup, Denmark; code No. S 203120 in distilled water, pH 6).

Immunohistochemistry was performed as described in detail previously with some slight modifications (Hannibal et al., 2014). The sections and the flat-mount retinas were incubated in 5% normal donkey serum for 20 min to avoid non-specific staining. Double or triple-immunostained sections were incubated overnight while the flat mount was incubated for 3 days at 4°C. The sections were washed and incubated with secondary antibodies at 60 min at room temperature or over-night at 4°C while the flat mount was incubated overnight at 4°C. Control experiments were performed by preabsorption of primary antibodies with their respective antigen or by eliminating the primary antibody, which abolished all specific staining.

### Photomicrography, cell counting, and morphological analysis

Images were obtained using an iMIC confocal microscope (Till Photonics, FEI, Germany) equipped with appropriate filter settings for detecting DAPI, CY2/Alexa488, Texas Red/Alexa561/594 and CY5/Alexa647. The iMIC was equipped with the following objectives: X20, numerical aperture (NA) = 0.75; X40, NA = 1.3 and X60, NA = 1.46. Using the X60, the highest resolution [(*r* = λ/NA), where λ is the imaging wavelength] was for X60 = 174 nm. Resolution in the z-axis were at X60 0.2 μm. All images in z-stacks were photographed using the X40 or X60 objective and all were deconvoluted in AutoQuantX, version 3.04 (Media Cybernetics, Inc. Rockville, USA) before analyzed in IMARIS® vers. 7.6 and 8.1 from Bitplane, Switzerland (http://www.bitplane.com). For the determination of co-localization of two markers in 3D images, the co-localization module in IMARIS® was used. Determination of co-localization between melanopsin and RBPMS in a single cell (2D) was done with a co-localization plug-in module in ImageJ/Fiji software (version. 1.47q, NIH, USA) in which the points of two 8-bit images with both antigens appeared white (we used default value = 255). Pixels were considered to reflect co-localization of the antigens if their intensities were higher than the threshold of their respective channels (we use threshold set at 50-100 depending on the background noise) and if the ratio of their intensity was higher than the ratio setting value (we used the default set at 50%).

**Figure 1:**
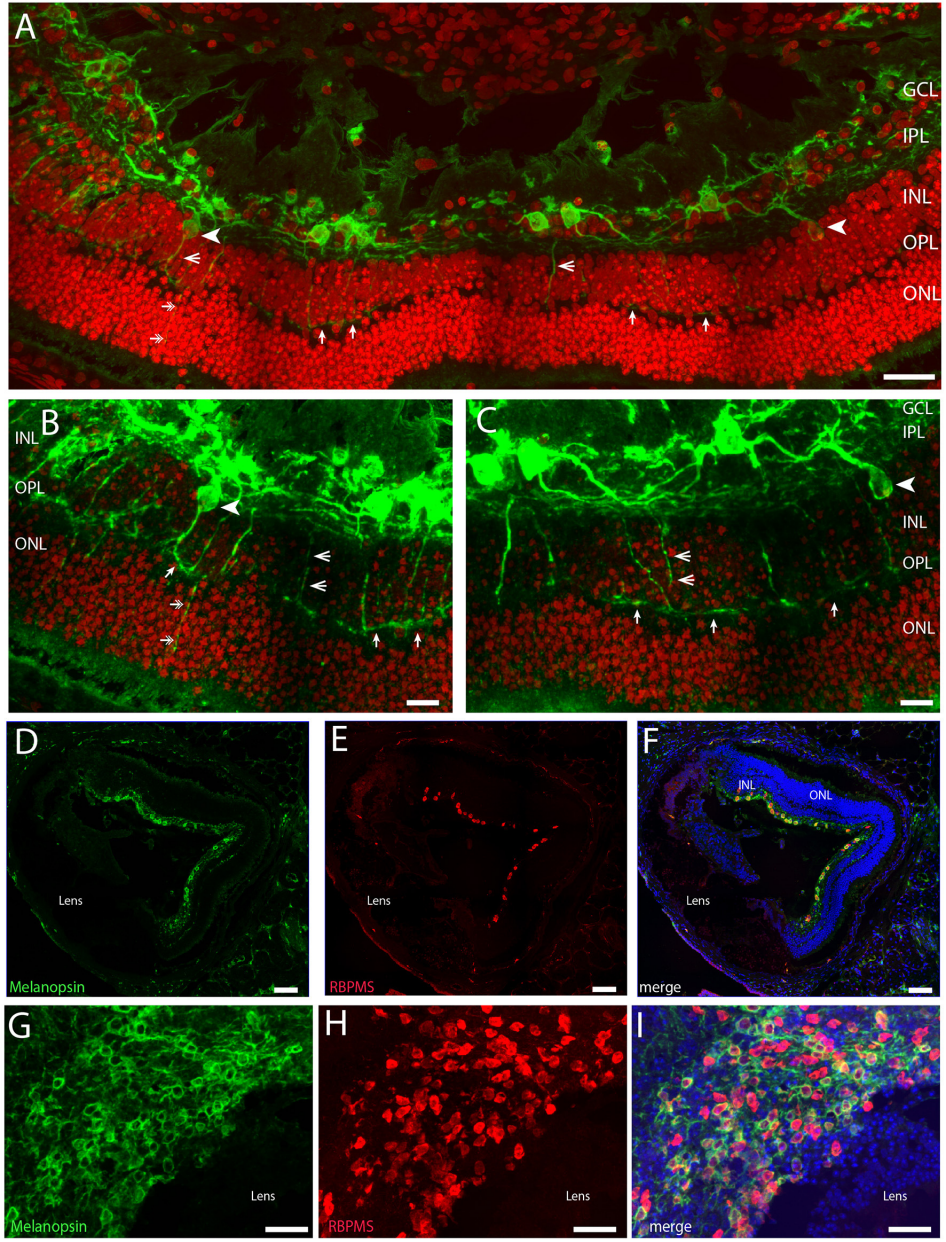
**Confocal photomicrographs of melanopsin (green) RGC (mRGCs) and DAPI nuclear counterstaining (red) in the ***Spalax*** retina (A–C)**. Melanopsin RGCs were located in the ganglion cell layer (GCL) and few displaced RGCs were found in the inner nuclear cell layer (INL) (indicated by arrowhead). mRGCs projected mainly into the IPL, but also to the outer plexiform layer (OPL) (exemplified by open arrowhead) where they formed an outer plexus (indicated by single arrows in **A–C**). Few processes were also seen in the outer nuclear layer (ONL) (indicated by double arrows in **A,B**). **(D–I)** Ganglion cell marker RBPMS (red) in combination with melanopsin (green) and DAPI nuclear counterstaining (blue) in sagittal sections **(D–F)** and horizontal sections through the GCL **(G–I)**. Scale bars: **(A)**; 40 μm, **(B,C)**; 15 μm, **(D–F)**; 100 μm, **(G–I)**; 50 μm.

**Figure 2:**
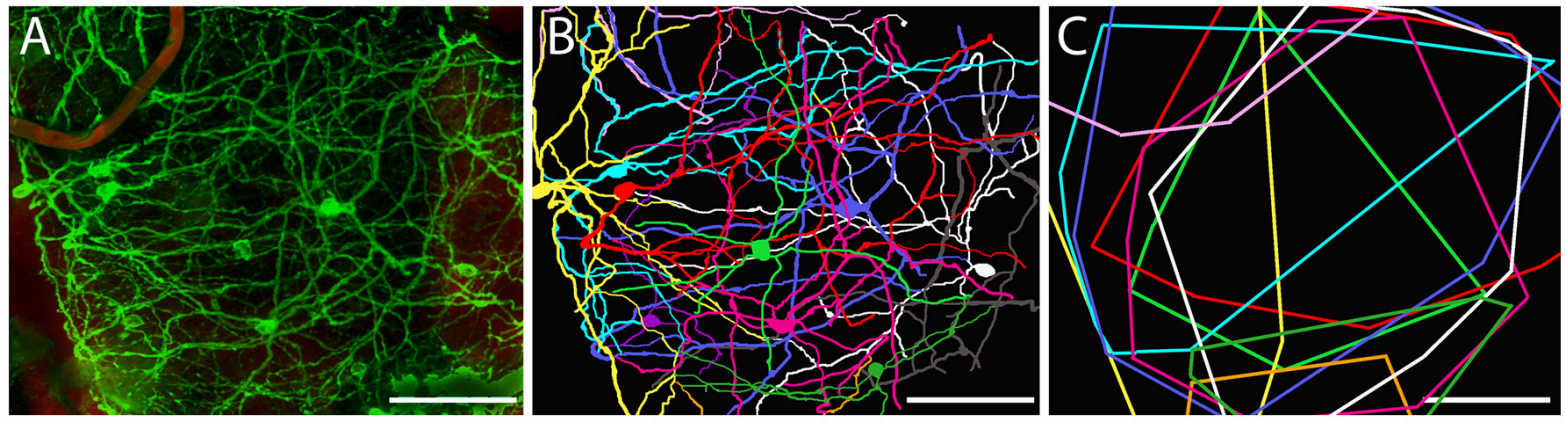
**Dendritic arborization of mRGCs in ***Spalax*** retina. (A)** Representative image from a region of Spalax whole-mount retina was reconstructed in 3D after labeling with anti-melanopsin antibody. **(B)** Representative drawings of the dendritic field and soma of mRGCs from the 3D reconstruction picture in **(A)**. **(C)** Minimal polygons fitted around the dendritic profile of each reconstructed mRGC in **(B)**. Note the complexity of the mRGCs dendritic plexus. Scale bar: 100 μm.

Melanopsin and RNA-binding protein with multiple splicing (RBPMS) cell counts were performed on every third section of a consecutive series of sections from one eye from two animals, which were photographed with the iMIC confocal microscope using X20 objective making Z stacks covering the thickness (12 micron, each section = 1 micron) (Figures 1D–F). Each z-stack taken of the retina were stitched together using the LA Stitch plug-in in Fiji software (version 1.47q, NIH, USA) to create an image of the entire retina. Each of these images were then analyzed as described above. In one animal, melanopsin/RBPMS cell density was also counted in sections cut horizontally through a large area of the ganglion cell layer (GCL) (Figures 1G–I) and the density was compared with the density of melanopsin cells obtained from sections and the calculated total cell count (see below). The flat mount retina was photomicrographed in the IMIC and a 3D reconstruction of Z-stacks (Z axis = 40 micron total) was created and analyzed in IMARIS® (after stitching all Z-stacks together using the LA Stitch plug-in in Fiji software (version 1.47q, NIH, USA)). Most likely due to strong attachment of the GCL and the degenerated lens, most of the GCL was lost. However, information of the density of displaced melanopsin RGCs and the melanopsin dendritic network in the IPL and OPL was confirmed and extended the observations from the retinal sections, and the density of displaced melanopsin RGCs could be estimated (see below and see Figure 2). To mesure dendritic areas of individual mRGCs, we traced the minimal convex polygon enclosing the dendritic field in 3D images of each labeled cell and we measured each dendritic profiles with the aid of Fiji software. Melanopsin expressing RGC subtypes were investigated and compared to the subtypes previously defined in mouse retina (Schmidt et al., 2011a; Cui et al., 2015), and the displaced melanopsin cells were counted using the 3D cell counting module in Fiji. The total cell count of melanopsin and RBPMS was presented with and without Abercrombie's correction (1946) (Guillery, 2002). For the correction factor we multiplied the counts in each section by T/T+h, where T is the section thickness (12 μm) and h is the mean height of the nuclear diameter. All images were adjusted for brightness and contrast either in Fiji or in Photoshop CS5 (Adobe, San Jose, CA) and mounted into plates in Adobe Illustrator CS5 (Adobe, San Jose, CA).

## Results

### Melanopsin RGCs stratify in both the inner and in the outer plexiform layers

The vertical sections from the *Spalax* eye demonstrated that the *Spalax* retina contains photoreceptor layer (ONL), outer plexiform layer (OPL), inner nuclear layer (INL), inner plexiform layer (IPL), and ganglion cell layer (GCL). These layers were less organized than in sighted mammals (Figure 1A) and examples of cells displaced from their localization in the seeing retina of nearly all cell types verified were found (see below).

**Figure 3:**
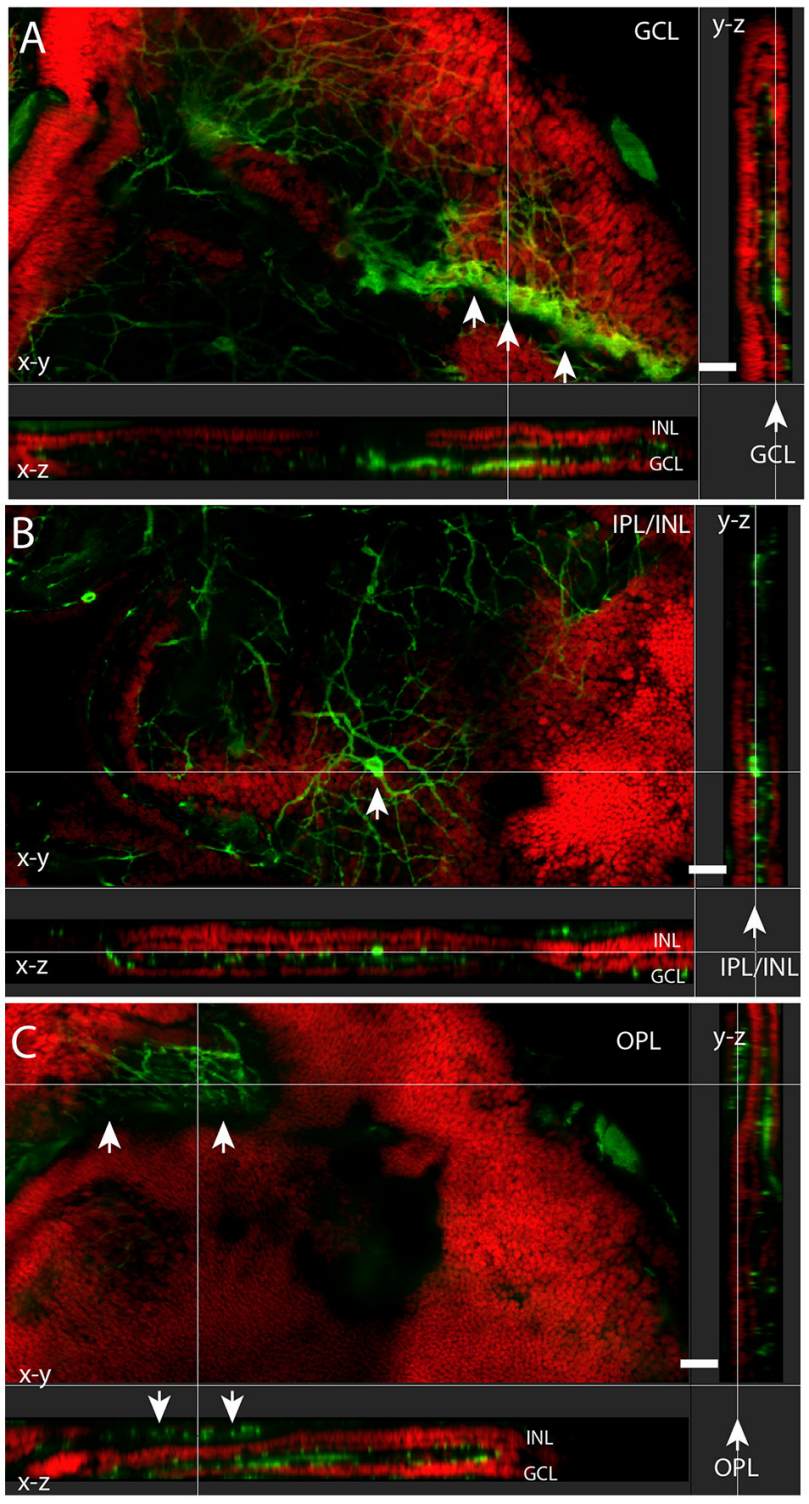
**Melanopsin (green) RGCs (mRGCs) and DAPI nuclear counterstaining (red) in flatmount ***Spalax*** retina**. XYZ images of the ganglion cell layer (GCL) **(A)**, inner plexiform/inner nuclear cell layer (IPL/INL) **(B)**, and outer plexiform layer (OPL) **(C)** showing melanopsin immunoreactivity in a minor rest of the GCL (arrows in **A**) and a displaced mRGC in the INL (arrow in **B**) and the melanopsin plexus in the OPL in **(C)** (indicated by arrows). Note the projections X-Z and Y-Z showing the different sublayers. Scale bars: **(A–C)**; 30 μm.

Approximately 95% of all melanopsin cells were located in the GCL and these mRGCs projected mainly into the IPL although dendritic processes were found in all sublayers of the IPL, forming a complex dendritic field (Figures 1A, 2). Nearly all mRGCs in the GCL sent dendrites to the OPL forming an outer plexus (Figures 1A–C). The highest density of dendrites were found close to the INL as in sighted retinas (Figures 1A–C, 3). Melanopsin cells located in the INL projected either to the IPL or into the outer plexus in the OPL (Figures 1B,C, 3C). Few melanopsin processes were also found in the ONL (Figures 1A–C). Despite the localization, no obvious difference was found between the mRGCs regarding soma size or dendritic branching or morphology although the cell density in the GCL makes it difficult to clearly discriminate dendritic profile (Figures 1G, 3A). The cell body typically had a diameter of 12.2 ± 0.4 μm and a dendritic field of 0.072 ± 0.006 mm^2^ with 3-4 dendritic main branches (Figures 2, 3B).

### Melanopsin is expressed in nearly 90% of all RGCs

Using the ganglion cell marker RBPMS (Rodriguez et al., 2014) in combination with melanopsin we found that the total number of mRGCs from two animals represents 890 ± 62 cells/retina. Of these, 87% (752 ± 40) contain melanopsin (cell density 788 melanopsin RGCs/mm^2^). We also counted the RBPMS and melanopsin positive cells in a horizontal section cut through the GCL (Figures 1G–I) with an area of 0.077 mm^2^. Using this preparation we found a total of 115 RBPMS containing cells. Of these, 73 RGCs co-stored melanopsin corresponding to a cell density of melanopsin RGCs of 949 cells/mm^2^, which is higher but in agreement with the cell counts obtained from the retinal sections. However, the population of non-melanopsin RBPMS positive cells was slightly higher in this section of the retina giving a total estimate of RGCs in the *Spalax* of 1495 RGCs/mm^2^. Overall the cell counting of RBPMS and melanopsin varied depending on the methods and tissue used but seems in agreement with the number of RGCs found by (Cooper et al., 1993a). He found ~900 RGCs in the *Spalax* retina identified using retrograde tract tracing from the exposed optic nerve (Cooper et al., 1993a). Cell counts made by counting RGCs in every third section from the two eyes may have resulted in “over-counting” (see Guillery, 2002). Therefore, we also corrected our cell count using the Abercrombie's correction (Guillery, 2002) using the mean diameter of the nucleus as described recently (La Morgia et al., 2015). Using the calculated Abercrombie correction factor [*T*/*T*+*h*, where *T* = 12 μm, nuclear average (*h*) = 8.69 μm] of 0.57, the total RGCs count reduced to 507 RGCs/retina. In comparison, the cell count of a section through the GCL of the “flatmount” was more reduced and may be underestimated by the correction.

**Figure 4:**
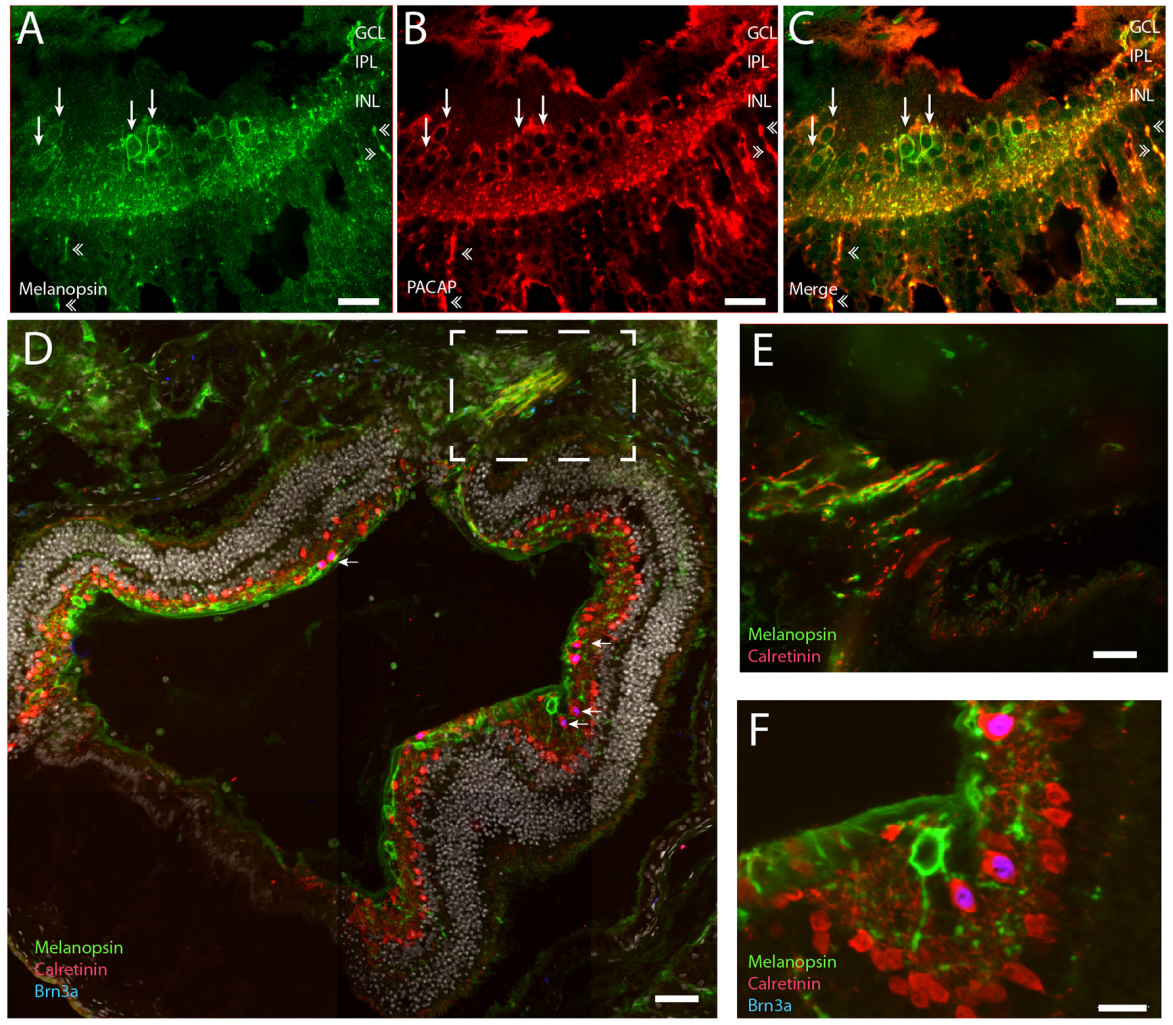
**Representative retinal section of PACAP (red) and melanopsin (green) co-expressed in mRGCs (A–C)**. Note that PACAP and melanopsin were found in RGCs (indicated by thin arrows in **B,C**) and in distal dendrites in the inner nuclear layer (INL) and outer plexiform later (OPL) (indicated by double arrowheads in **A–C**). Calretinin (red) was found in the subpopulation of non-melanopsin RGCs and in amacrine cells in the INL. Brn3a (blue) was found in all non-melanopsin RGCs co-storing calretinin **(D–F)** (nuclear counterstaining with DAPI in gray in **D**). Panel **(E)** shows the frame indicated in **(D)** of the optic nerve containing melanopsin and calretinin positive axons. Panel **(F)** Shows Brn3a in non-mRGCs in high magnification. Scale bars: **(A–C)**; 30 μm, **(D)**; 50 μm, **(E,F)**; 15 μm. GCL; ganglion cell layer, IPL; inner plexiform layer, INL; inner nuclear layer.

We also counted displaced melanopsin cells in one flat mount retina and found 19 cells/0.49 mm^2^. If the retina is ~1.3 mm^2^ (Cooper et al., 1993a) this corresponds to ~50 displaced melanopsin cells in the whole *Spalax* retina. This gives a ratio of ~5% of the total number of mRGCs displaced in the INL. As previously reported (Hannibal et al., 2002b) we found PACAP in all melanopsin expressing RGCs and in dendrites located in both IPL and OPL and in the few melanopsin processes in ONL (Figures 4A–F).

### Non-melanopsin RGCs express Brn3A and calretinin

We investigated whether the remaining non-melanopsin RGCs could be classified by known ganglion cell markers (Lee et al., 2010; Jain et al., 2012; Nadal-Nicolas et al., 2012). We found that all non-melanopsin RGCs (138 ± 22) costored Brn3a and calretinin (Figures 4D–F). While Brn3a had a nuclear localization, calretinin was found in the cytoplasm and in the neuronal processes including axons. In sections which the optic nerve leaves the eye, both melanopsin—and calretinin containing axons could be demonstrated (Figures 4D,E).

### Melanopsin dendrites are found in close apposition with rods and cones pedicles suggesting synaptic contacts

**Figure 5:**
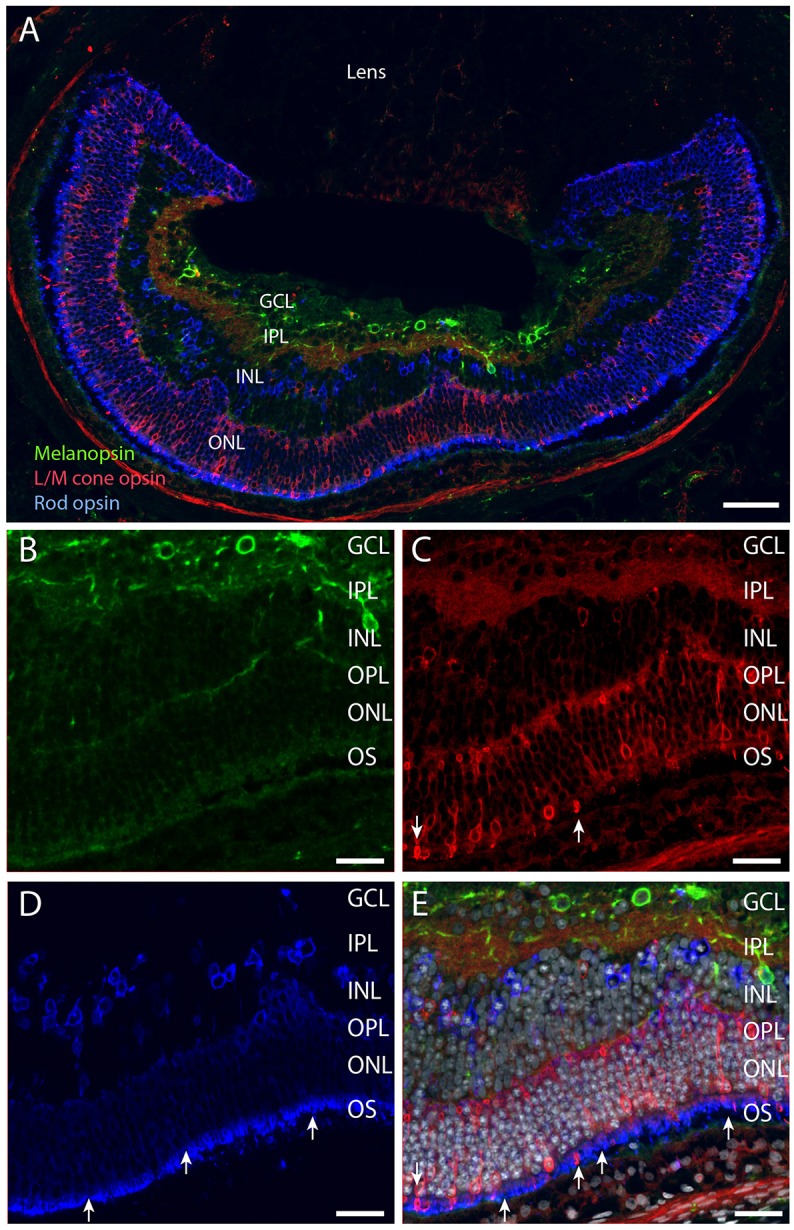
**Melanopsin (green), L/M cone opsin (red) and rhodopsin (blue) and DAPI counter staining (gray) in ***Spalax*** (A–E)**. Melanopsin was expressed in the RGCs located in the GCL and in the INL **(B)**. L/M cone opsin was expressed in photoreceptor cells in the ONL, and displaced in the INL **(A,C,E)**. Rhodopsin was expressed in photoreceptor cells located in the ONL and in displaced cells in the INL **(A,D,E)**. The strongest immunostaining in both rods and cones was found in the degenerated outer segment (arrows in **C–E**). Scale bars: **(A)**; 70 μm, **(B–E)**; 35 μm. GCL; ganglion cell layer, IPL; inner plexiform layer, INL; inner nuclear cell layer, OPL; outer plexiform layer, ONL; outer nuclear cell layer, OS; outer (photoreceptor) segment.

**Figure 6:**
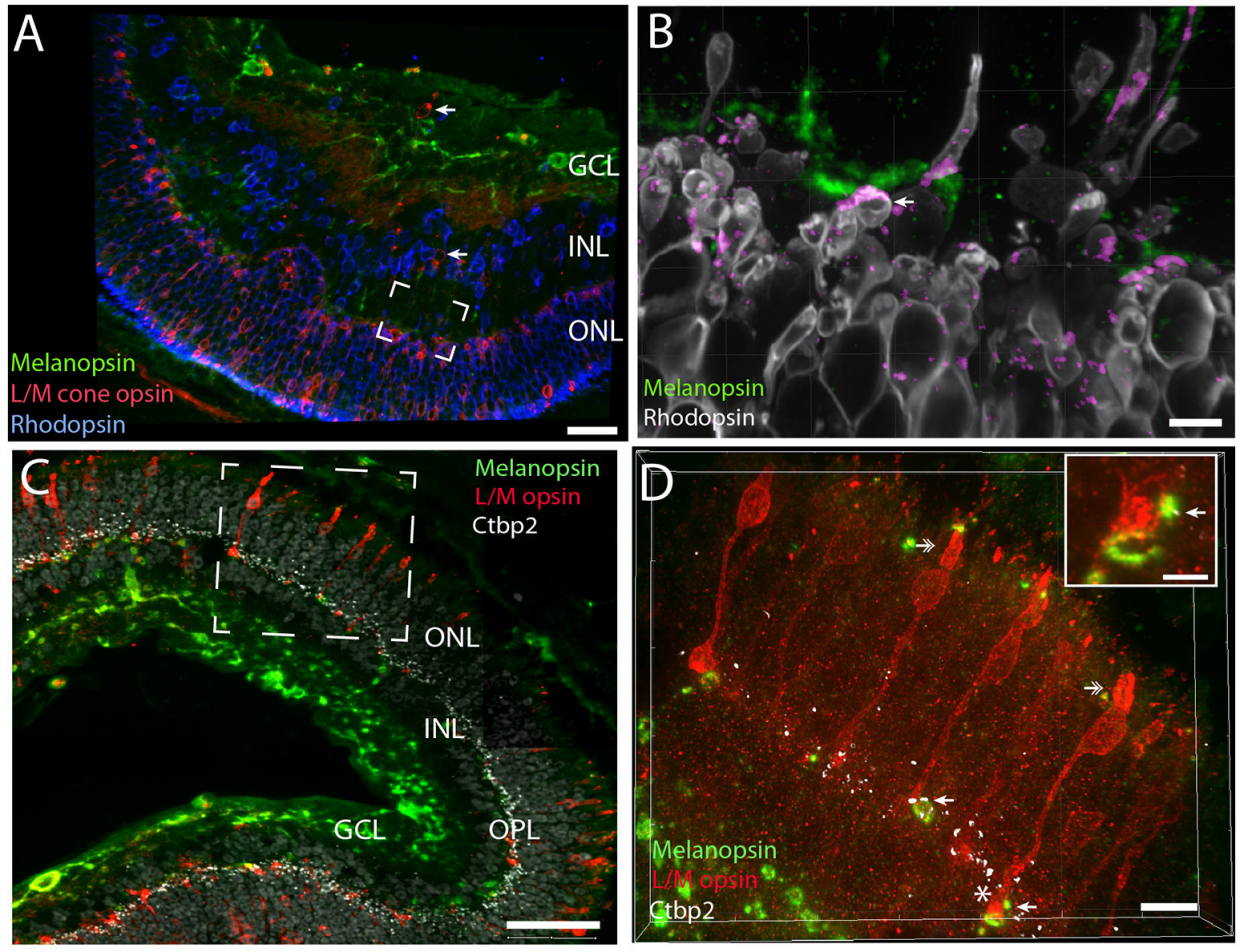
**Melanopsin dendrites were found in close apposition with rods and cones indicating synaptic contact**. Melanopsin (green), L/M cone opsin (red) and rhodopsin (blue/gray) stainings in *Spalax* **(A)**. Rod and cone opsins were expressed in the cell membrane of the soma, in the inner segment and in the outer segments **(B–D)**. In the OPL melanopsin dendritic processes are found in close apposition with rod pedicles (framed area in **A** is shown in high power in **B**) and co-localization indicating synaptic contacts (determined by IMARIS® colocalization module) as shown in purple. Ribbon containing synapses visualized by Ctbp2 (white in **C,D** can be found in L/M-cone pedicles of the OPL). Likely synaptic contact between L/M cone pedicles and melanopsin distal processes can be found in the OPL. The frame area in C shown L/M cone opsin photoreceptor cells costoring Ctbp2. By using IMARIS colocalization module overlap of L/M cone opsin, CtBp2, and melanopsin are indicated by arrows in **(D)** and in the insert in Panel **(D)** (yellow dots corresponding to the area in **C** indicated by ^*^). Double arrows in **(D)** show the degenerated outer segment in two L/M-cone photoreceptor cells. The grid in Panel **(B–D)** indicates that the image was created in 3D. Scale bars: **(A)**; 40 μm, **(B)**; 5 μm, **(C)**; 50 μm, **(D)**; 10 μm and insert in **(D)**; 4 μm. INL; inner nuclear cell layer, OPL; outer plexiform layer, ONL; outer nuclear cell layer.

**Figure 7:**
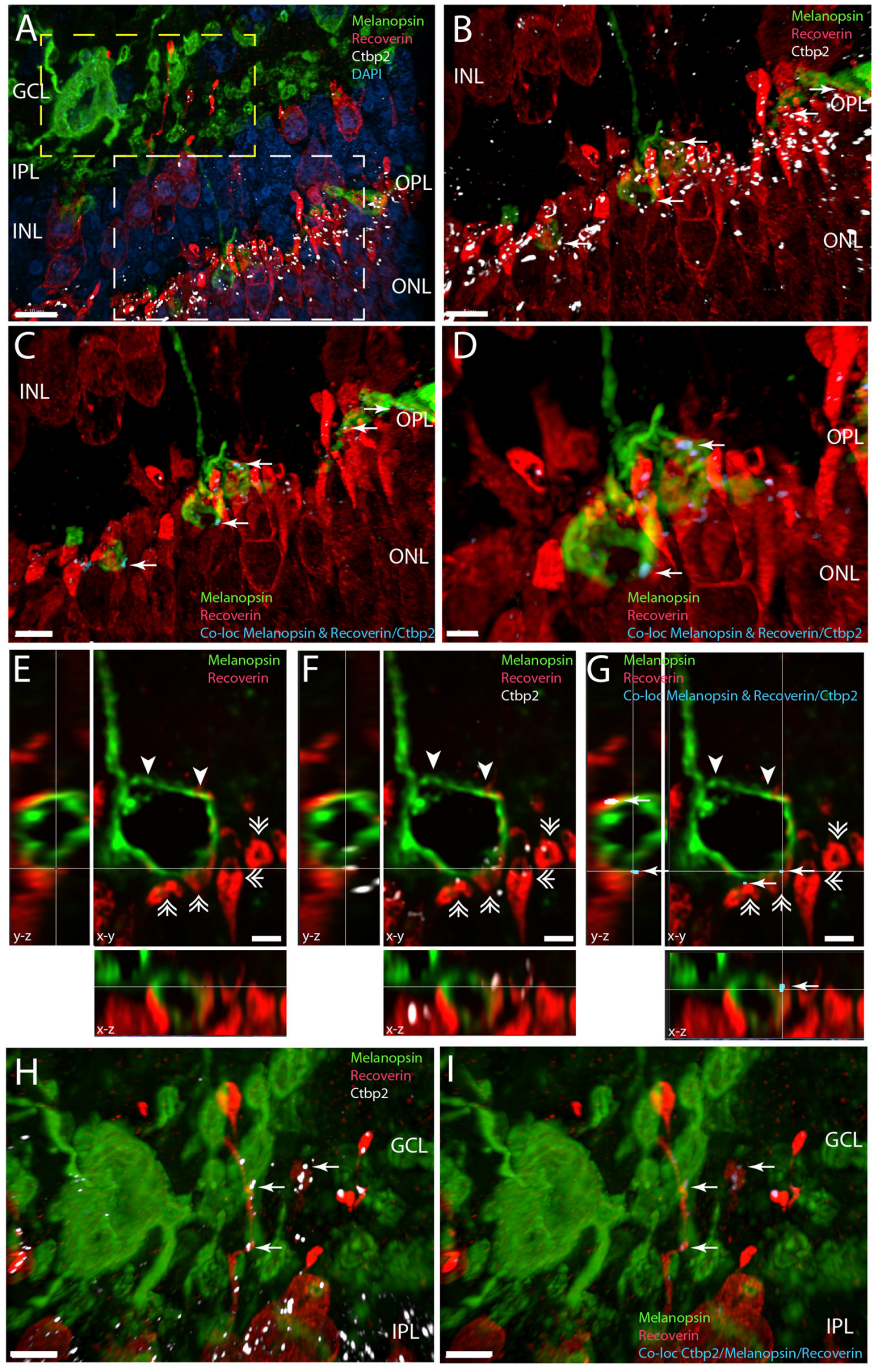
**Melanopsin (green) RGCs are innervated by recoverin (red) expressing bipolar cells in the IPL/GCL (yellow frame in A and in higher magnification in H,I) and have direct synaptic contact with recoverin containing photoreceptor cells in the OPL expressing Ctbp2, a marker for synaptic ribbons (white) (white frame in A and ultra-high magnification in C,G)**. Panels **(A–D)** and panels **(H,I)** are 3D reconstructions representing 65 sections separated by 0.2 μm. For better illustration, single digital section **(E–G)** representing x-y, x-z, and y-z views are shown of the distal melanopsin process (indicated by arrowhead in **E,G**) and contact with recoverin containg photoreceptor pedicles (indicated by double arrow heads) also containing the synaptic marker Ctbp2 (shown in white in **F**). The synaptic contacts in which melanopsin have direct overlap with the co-localization of recoverin/Ctbp2 are indicated by arrows in blue **(C–G)**. Synaptic contact between melanopsin and recoverin containing bipolar terminals co-storing Ctbp2 are indicated by arrows in blue in I. Scale bars: **(A)**; 10 μm, **(B,C)**, and **(H,I)**; 5 μm, **(D)**, **(E–G)**; 2 μm.

The *Spalax* retina has been shown to be rich in rhodopsin expressing cells located in the ONL but also in the INL (Janssen et al., 2000). In contrast, only functional studies have indicated the occurrence of long wave sensitive photoreceptor bearing cells in the *Spalax* retina (David-Gray et al., 1999; Janssen et al., 2003). We found strong rhodopsin labeling in cells of the ONL (Figures 5A,D). The most intense staining was located in a degenerated outer segment, but staining was also found in the membrane of the soma of the photoreceptor cells (Figures 5A,D,E, 6). Rhodopsin immunoreactive cells were also found in the INL, what seems to be in displaced photoreceptor cells (Figures 5, 6). L/M cone opsin immunoreactivity was demonstrated in another population of photoreceptor cells, primarily located in the ONL but also in few displaced cells in the INL (Figures 5A,C,E, 6A,C,D). Compared to rhodopsin cells, the number of L/M cone opsin expressing cells were fewer in both the ONL and in the INL (Figures 5, 6). The L/M cone opsin photoreceptor cells showed most intense immunoreactivity in the outer segment (Figures 6C,D) but L/M opsin was also seen in the cell membrane of the soma and the inner segment (Figure 6D). The L/M cone opsin positive cells located in the INL seem to lack the outer segment and projected into the IPL or GCL (Figures 5C,E, 6A). All melanopsin containing dendrites penetrating into the OPL were found in close opposition to both rod—and cone pedicles (Figures 6B–D, 7). 3D-reconstruction of melanopsin dendrites demonstrated large dendritic terminal synaptic bouttons up to 3–4 μm in diameter (Figure 6). The terminals were found in close contact with rod pedicles (Figure 6B). Co-localization analysis demonstrated pixel overlap of the two antigens indicating direct synaptic contact between rod pedicles and melanopsin distal processes in the OPL (Figure 6B). Melanopsin distal dendrites were also found in close apposition to L/M cone pedicles (Figures 6C,D). Within the cone pedicles the synaptic ribbon marker Ctbp2 was found (Figure 6D). Co-localization analysis after 3D reconstruction revealed direct synaptic contact between melanopsin containing processes, L/M cone pedicles, and the synaptic ribbon marker Ctbp2 (Figure 6D).

Recoverin is found in photoreceptor cells and in subtypes of cone bipolar cells (Euler and Wassle, 1995). In the *Spalax* we also found recoverin in photoreceptor bearing cells in both the ONL and INL (Figure 7). 3D analysis confirmed that recoverin positive photoreceptor cell pedicles costore Ctbp2 immunoreactivity (Figures 7A,B,F), which was found in close apposition to distal melanopsin processes in the OPL indicating synaptic contact (Figure 7). This is demonstrated on stacks of digital sections used for 3D reconstructions and co-localizations analysis as shown in Figure 7. In the INL recoverin staining most likely stained displaced cone opsin expressing cells and bipolar cells (Figure 7). The recoverin positive cells located in the inner part of the INL had their processes located in the IPL and close to melanopsin somata and processes. 3D analysis demonstrated distinct location of Ctpb2 immunoreactivity in bipolar terminals in close apposition to melanopsin processes indicating direct synaptic contact with recoverin positive bipolar cells and melanopsin processes in the IPL/GCL (Figures 7A,H,I).

We also used an antibody directed against mammalian S-cones (Table 1). This antibody did not reveal any specific staining in agreement with a previous study (David-Gray et al., 2002). Previously, this antibody has been shown to immunolabel S-cones in humans (Milam et al., 2002), mice (Dkhissi-Benyahya et al., 2006), ground squirrels (Sakai et al., 2003), and in *Talpa occidentalis* (Carmona et al., 2010).

### Calretinin expressing amacrine cells innervate melanopsin RGCs

**Figure 8:**
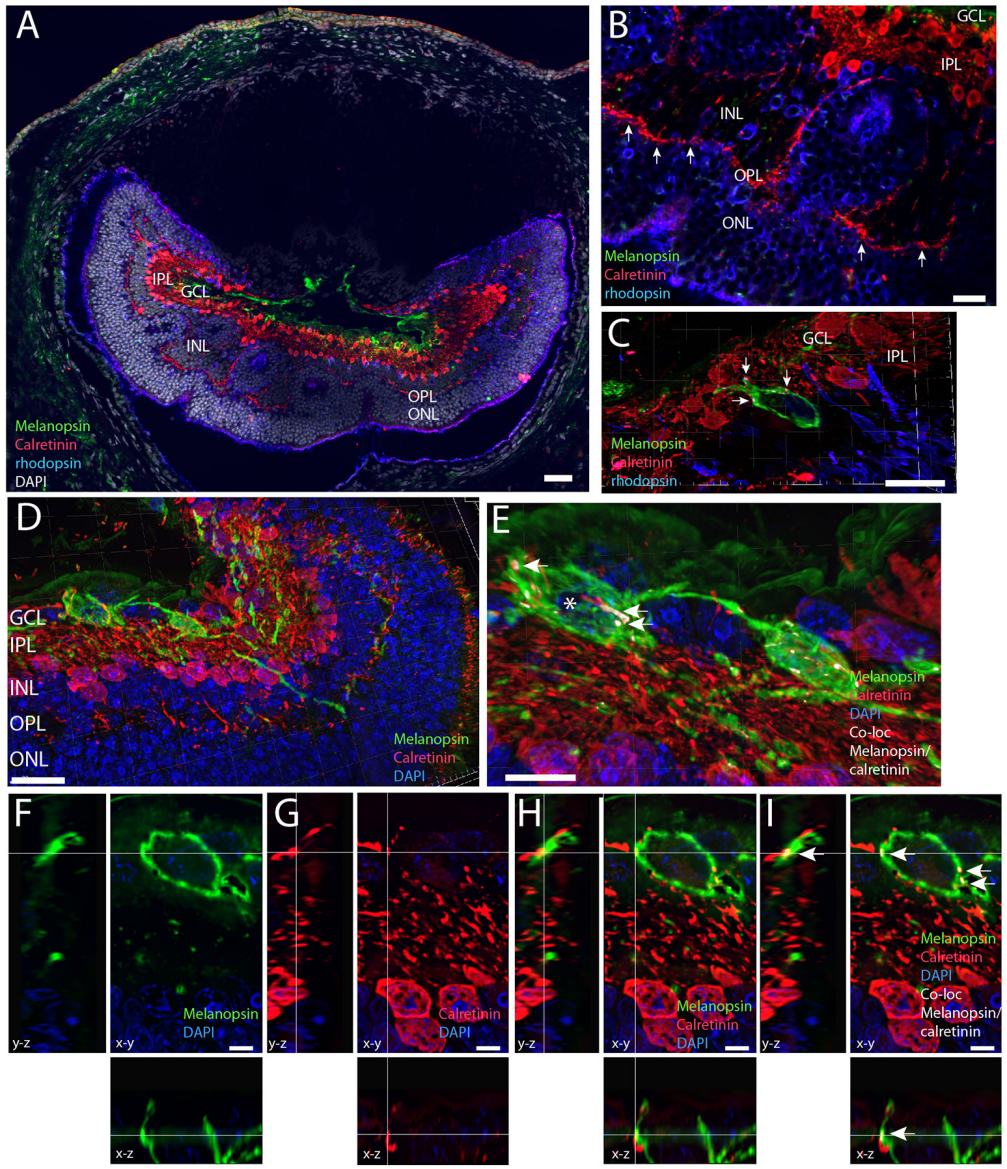
**Melanopsin (green) RGCs are innervated by calretinin expressing amacrine cells (red). (A)** Calretinin immunoreactivity in the ***Spalax*** was found in amacrine cells in the inner part of the INL and in cells of the outer part of the INL, which could be horizontal cells and in processes innervating the OPL in close contact with rod photoreceptors (blue). In the IPL/GCL calretinin processes have close contact to melanopsin processes. Using the IMARIS co-localization module on 73 digital sections separated by 0.2 μm used for 3D reconstruction co-localization indicating synaptic contact was found between melanopsin soma and proximal processes and calretinin axons. Panels **(D,E)** are 3D images and for better illustration, single digital section **(F–I)** representing x-y, x-z, and y-z views are shown of the melanopsin cell indicated by ^*^ as well as single channels. The calculated co-localization of melanopsin and calretinin in I indicated by arrows. Scale bars: **(A)**; 50 μm, **(B)**, and **(D)**; 20 μm, **(C)**; 15 μm, **(E)** and **(G)**; 10 μm., **(F–I)**; 5 μm. GCL; ganglion cell layer, IPL; inner plexiform layer, INL; inner nuclear cell layer, OPL; outer plexiform layer, ONL; outer nuclear cell layer.

Calretinin has been shown in different subtypes of retinal cells in the mammalian retina including ganglion cells, amacrine and horizontal cells (Lee et al., 2010, 2016). Calretinin immunolabeling was found in the INL located in the outer part of the INL most likely representing horizontal cells (Figure 8A). A larger contingent of calretinin expressing cells was found in the inner part of the INL most likely representing a subpopulation of amacrine cells (Figures 8A–D), and in ganglion cells in cells which co-store Brn3a (see above). Calretinin located in amacrine cells was found in the cell cytoplasm and in the dendritic processes in the IPL and OPL (Figure 8). Many of the processes penetrate the INL and terminate in the OPL in close apposition to rod and L/M cone pedicles (Figures 8A,B). Other processes were found in the IPL with no sublayer organization as seen previously (Cernuda-Cernuda et al., 2002). 3D reconstruction and analysis revealed calretinin containing processes of the IPL and GCL were in close apposition with melanopsin dendrites and soma membrane most likely making synaptic contacts (Figures 8E–I).

PKC-α which is recognized as a valid marker of rod bipolar cells in seeing eyes (Greferath et al., 1990) was not convincingly demonstrated in the *Spalax* retina despite using several different antibodies raised against PKC- α most likely due to species difference of PKC in *Spalax*.

## Discussion

The present study extends previous observations in the *Spalax*, an animal which adapted anatomy and physiology to a blind subterranean life and developed subcutaneous eyes with the ability to sense environmental light for circadian and photoperiodic regulation (Sanyal et al., 1990; Cooper et al., 1993a,b; David-Gray et al., 1998). The eyes of the *Spalax*, although severely degenerated with no visual capacity and with a degenerated reorganized retina, expresses three types of photoreceptors, melanopsin, rhodopsin, and L/M cone opsin. The majority (87%) of the central projections are melanopsin expressing RGCs while the remaining RGCs express the transcription factor Brn3a and calretinin. The sighted eye converts light information from rod and cones via bipolar cells to the GCLs. The *Spalax* retina seems to be wired differently. In the *Spalax*, direct contacts between rods, cones, and melanopsin containing dendrites most likely represents synaptic contacts, are demonstrated in addition to input from calretinin containing amacrine cells and cone bipolar cells. These observations propose the *Spalax* retina to be a possible model for research of NIF system.

We used a large number of antibody markers, which have been characterized in the sighted retina and found that most of the applied antibodies have the same specificity in the *Spalax* retina. We were unable to identify dopaminergic or VIP-ergic expressing amacrine cells, although both antibodies stain neurons and nerve fibers in the *Spalax* central nervous system, indicating a lack of VIP and dopaminergic amacrine cells in the *Spalax* retina. Neither could we find any evidence for short wave opsin expressing photoreceptors in the *Spalax* as previously found in other subterranean species like the *Talpa occidentalis* (Carmona et al., 2010). We cannot exclude the existence of the above mentioned cell systems, but it is likely that the evolutionary selection on the light detection system of the *Spalax* can explain lack of these systems.

### Two distinct types of RGCs are found in the *Spalax* retina

The morphology of the *Spalax* retina has been examined at both the light and electronical level (Cernuda-Cernuda et al., 2002). Previously, we identified melanopsin in a large group of RGCs, which co-express the neuropeptide PACAP, a neurotransmitter in the retinohypothalamic tract innervating the SCN (Hannibal et al., 2002b). Here we provide a more detailed analysis of mRGCs and demonstrate that in the *Spalax* retina, mRGCs constitute the major type of RGCs (87%) with cell bodies located in both the GCL and displaced in the INL, similar to the sighted retina. In the *Spalax*, we were unable to classify mRGCs as in the mouse and rat retinas (Schmidt and Kofuji, 2009; Schmidt et al., 2011b; Esquiva et al., 2013; Reifler et al., 2015). However, subtypes may exist since the differently located mRGCs are wired differently with cell bodies in the GCL and INL and a different stratification of dendritic processes. In the sighted retina, mRGCs are involved in the regulation of the pupillary light reflex, which does not exist in the *Spalax* (Schmidt et al., 2011b). Melanopsin is also not involved in visual tasks as suggested in the sighted retina (Allen et al., 2014; Storchi et al., 2015).

Total cell count using the RGC marker RBPMS (Rodriguez et al., 2014) reveals that ~900 RGCs are found in the *Spalax* retina depending on the method used for cell counting. This is very similar to that found in a previous study by Cooper et al. (1993a) made on flat mount retina after retrograde tracing from the optic nerve. We provided central profile counting (La Morgia et al., 2015) on sections cut sagittally through the entire eye. We applied a “section separation” approach by counting every 3rd section to minimize counting errors. Due to the possibility of “over counting” in the sagittal sections, we also provided a corrected number using the equation by Abercrombie (Guillery, 2002). Although, this method should ensure the problems of over-counting, compared to the cell counts obtained in the horizontal section, we may have underestimated the total number of RGCs after Abercrombies correction. Despite the discrepancy in mRGCs counts depending on the material used, 87% express melanopsin and of these 5% were displaced in the INL. The amount of displaced mRGCs is less than in sighted eyes although the fraction of displaced cells differs between mammalian species. In humans close to 50% of all melanopsin cells are displaced (Dacey et al., 2005), in nocturnal rodents like rat and mice (Nadal-Nicolas et al., 2014; Valiente-Soriano et al., 2014) ~5–10% were displaced, while in the diurnal grass rat, *Arvicanthis niloticus*, 25% of the melanopsin RGCs are displaced into the INL (Langel et al., 2015). In the *Talpa occidentalis*, another blind mole rat, mRGCs were also reported to be the dominant type of RGCs although no information are available on the number of displaced mRGCs, or on mRGC density (Carmona et al., 2010). So far, no functional role has been attributed to the different distribution of displaced mRGCs, but it is likely, that these cells receive different synaptic inputs from the retina which represents functional difference of the mRGCs (circadian entrainment, pupillary light reflex, nocturnal suppression of melatonin, masking behavior).

We also identified another population of RGCs in the *Spalax* retina. This subpopulation was characterized by the expression of Brn3a, calretinin and RBPMS. Brn3a has been shown to co-localize with RBPMS in mouse, whereas a subpopulation of mRGCs express Brn3b, but not Brn3a (Galindo-Romero et al., 2013; Nadal-Nicolas et al., 2014; Rodriguez et al., 2014). In the *Spalax*, Brn3a may represent a subpopulation of the Brn3a positive RGCs found in the sighted eye. Central retinal projections in the *Spalax* innervate several areas in the brain, the most dense projection reaches the SCN, but several areas in the for—and midbrain are also innervated from the eyes (Bronchti et al., 1991; Cooper et al., 1993b). It remains to be shown, whether Brn3a expressing RGCs in the *Spalax* are targeting the SCN and involved in circadian timing, or whether these projections reach other areas involved in other NIF related functions. It is interesting that in sighted animals several studies have demonstrated non-melanopsin retinal projections in the SCN, some which may represent Brn3a expressing RGCs (Hannibal and Fahrenkrug, 2004; Hattar et al., 2006; Hannibal et al., 2014).

### Melanopsin RGCs organization and synaptic contact with rods and cones

Melanopsin RGCs can in the sighted eye be characterized by their stratifying processes as inner and outer stratifying cells (Dacey et al., 2005; Schmidt and Kofuji, 2009) and electron microscopy (EM) studies have shown that the mRGCs receive input from bipolar and amacrine cells in stratum 5 close to the INL and stratum 1 close to the GCL (Belenky et al., 2003). In the *Spalax*, input seems to originate directly from both the rods and cones and from bipolar and amacrine cells. Melanopsin dendrites stratify in both the IPL layer as found in the sighted eye, but also in the OPL and seems to have direct contact to both rod and cone pedicles. This is a significant difference compared to melanopsin projections in the seeing retina (Schmidt et al., 2011a). We used high magnification confocal light microscopy with a resolution of ~200 nm and identified potential synapses between rods, cones, and melanopsin by using the synaptic ribbon marker Ctbp2 (Schmitz et al., 2000; Sterling and Matthews, 2005) in combination with 3D analysis and computer based co-localization analysis. One EM study demonstrated that rod/cone pedicles in the *Spalax* contain ribbon synapses, as found in the sighted retina. Such synapses were found close to inner photoreceptor segments as well in the OPL (Cernuda-Cernuda et al., 2002). Our approach reveals close contact between melanopsin distal dendrtites and rod and cone pedicles with co-localization of Ctbp2 most likely representing synaptic contacts. In the study by Cernuda-Cernuda et al. (2002) rhodopsin expressing photoreceptors were identified at electronmicroscopy level (EM). Many of the photoreceptor pedicles located in the OPL contain synaptic ribbons and some were also located in the INL. We found rhodopsin immunoreactivity to be located in the outer (degenerated) segment and in the membrane of rod photoreceptor bearing cells, some of which were displaced in the INL. This is in agreement with observation by Cernuda-Cernuda et al. (2002). For the first time we identified L/W cone opsin expressing photoreceptor. The cone photoreceptor cells were as the rods located mainly in the ONL but were fewer in numbers than rods and few cone opsin bearing cells were found displaced to the INL. Our findings provide the anatomical substrate for functional occurrence of a green cone-like pigment reported previously (Janssen et al., 2003). This rewiring of melanopsin processes may increase and strengthen light information to the melanopsin RGCs. S cone-opsin was not found by immunohistochemistry in our work, supporting the hypothesis that *Spalax* lacks the UVS/VS cone photopigment (David-Gray et al., 2002). However, S-opsin occurs in the African mole rat (*Rodentia Bathyergidae*) (Peichl et al., 2004; Nemec et al., 2008) and in the European mole rats (*Talpa europaea*) (Glosmann et al., 2008), both having superficial eyes vs the subcutaneous eye in *Spalax*.

### Melanopsin RGCs are innervated by calretinin expressing amacrine cells and cone bipolar cells

In addition to light input information from the outer retina, the mRGCs also receive input through amacrine and bipolar cells in the *Spalax*. Previous EM studies in the sighted retina have provided evidence for such inputs, some of which are via ribbon synapse (Belenky et al., 2003). By using a ribbon marker and high resolution 3D analysis we found ribbon synapses in recoverin bipolar cells which were in close contact with melanopsin processes in the IPL most likely representing synaptic contacts. Recoverin is found in photoreceptor cells and subtypes of cone bipolar cells (Euler and Wassle, 1995). Our findings support synaptic contacts between photoreceptor cells expressing recoverin in the OPL. Although, we cannot differentiate between different subtypes of cone bipolar cells it seems recoverin positive cone bipolar cells with ribbon synapses make synaptic contacts with mRGCs dendrites in the OPL/GCL. We cannot exclude that the recoverin positive cells in the INL could be displaced cone photoreceptor cells, but their localization makes it more likely to be bipolar cells. The functional significance of these inputs to the melanopsin RGCs remain to be determined. Color information was recently shown to be mediated by melanopsin RGCs (Walmsley et al., 2015). Since the *Spalax* eyes are hidden behind fur, skin and buried in the harderian gland, it seems likely that other wavelengths than blue light of 490 nm, via rods and L/M cones can influence the output of the melanopsin system to the brain.

We found no convincing staining of PKC, which is recognized as a valid marker of rod bipolar cells (Greferath et al., 1990). We used several different antibodies and found only weak staining in cells the INL but due to low signal intensity, it was not possible to determine cell type or projections (not shown). Although, the existence of input to mRGCs from rod bipolar cells has been demonstrated in monkey (Jusuf et al., 2007) and in rats (Ostergaard et al., 2007), it remains to be shown to exist in the *Spalax* retina.

In conclusion, our study demonstrates the complex retinal circuitry used by the *Spalax* to detect light using rhodopsin, L/W cone opsin and melanopsin, provides evidence that the major projections to the brain (87%) are mediated via mRGCs and a non-melanopsin projecting pathway to the brain exists in the *Spalax*.

## Author contributions

JH developed the idea of the study. AA delivered the animals. JH and GE performed the IHC part and wrote the first draft. JH and GE made the figures and the Supplementary Movies from images obtained on the iMIC. GE performed the cell counting and measured the dendtritic field. GE and JH finalized the manuscript.

### Conflict of interest statement

The authors declare that the research was conducted in the absence of any commercial or financial relationships that could be construed as a potential conflict of interest.

## References

[B1] Allen A. E. Storchi R. Martial F. P. Petersen R. S. Montemurro M. A. Brown T. M.. (2014). Melanopsin-driven light adaptation in mouse vision. Curr. Biol. 24, 2481–2490. 10.1016/j.cub.2014.09.015 25308073 PMC4228053

[B2] Belenky M. A. Smeraski C. A. Provencio I. Sollars P. J. Pickard G. E. (2003). Melanopsin retinal ganglion cells receive bipolar and amacrine cell synapses. J. Comp. Neurol. 460, 380–393. 10.1002/cne.10652 12692856

[B3] Bronchti G. Rado R. Terkel J. Wollberg Z. (1991). Retinal projections in the blind mole rat: a WGA-HRP tracing study of a natural degeneration. Brain Res. Dev. Brain Res. 58, 159–170. 10.1016/0165-3806(91)90002-Z 2029763

[B4] Carmona F. D. Glosmann M. Ou J. Jimenez R. Collinson J. M. (2010). Retinal development and function in a 'blind' mole. Proc. Biol. Sci. 277, 1513–1522. 10.1098/rspb.2009.1744 20007180 PMC2871828

[B5] Cernuda-Cernuda R. DeGrip W. J. Cooper H. M. Nevo E. Garcia-Fernandez J. M. (2002). The retina of Spalax ehrenbergi. Novel histological features supportive of a modified photosensory role. Invest. Ophthalmol. Vis. Sci. 43, 2374–2383. 12091440

[B6] Cooper H. M. Herbin M. Nevo E. (1993a). Ocular regression conceals adaptive progression of the visual system in a blind subterranean mammal. Nature 361, 156–159. 7678449 10.1038/361156a0

[B7] Cooper H. M. Herbin M. Nevo E. (1993b). Visual system of a naturally microphthalmic mammal: the blind mole rat, Spalax ehrenbergi. [Review]. J. Comp. Neurol. 328, 313–350. 8440785 10.1002/cne.903280302

[B8] Cui Q. Ren C. Sollars P. J. Pickard G. E. So K. F. (2015). The injury resistant ability of melanopsin-expressing intrinsically photosensitive retinal ganglion cells. Neuroscience 284C, 845–853. 10.1016/j.neuroscience.2014.11.002 25446359 PMC4637166

[B9] Dacey D. M. Liao H. W. Peterson B. B. Robinson F. R. Smith V. C. Pokorny J.. (2005). Melanopsin-expressing ganglion cells in primate retina signal colour and irradiance and project to the LGN. Nature 433, 749–754. 10.1038/nature03387 15716953

[B10] David-Gray Z. K. Bellingham J. Munoz M. Avivi A. Nevo E. Foster R. G. (2002). Adaptive loss of ultraviolet-sensitive/violet-sensitive (UVS/VS) cone opsin in the blind mole rat (Spalax ehrenbergi). Eur. J. Neurosci. 16, 1186–1194. 10.1046/j.1460-9568.2002.02161.x 12405979

[B11] David-Gray Z. K. Cooper H. M. Janssen J. W. Nevo E. Foster R. G. (1999). Spectral tuning of a circadian photopigment in a subterranean ‘blind’ mammal (Spalax ehrenbergi). FEBS Lett. 461, 343–347. 10.1016/S0014-5793(99)01455-6 10567724

[B12] David-Gray Z. K. Janssen J. W. DeGrip W. J. Nevo E. Foster R. G. (1998). Light detection in a ‘blind’ mammal. Nat. Neurosci. 1, 655–656. 10.1038/3656 10196579

[B13] Dkhissi-Benyahya O. Rieux C. Hut R. A. Cooper H. M. (2006). Immunohistochemical evidence of a melanopsin cone in human retina. Invest. Ophthalmol. Vis. Sci. 47, 1636–1641. 10.1167/iovs.05-1459 16565403

[B14] Esquiva G. Lax P. Cuenca N. (2013). Impairment of intrinsically photosensitive retinal ganglion cells associated with late stages of retinal degeneration. Invest. Ophthalmol. Vis. Sci. 54, 4605–4618. 10.1167/iovs.13-12120 23766478

[B15] Euler T. Wassle H. (1995). Immunocytochemical identification of cone bipolar cells in the rat retina. J. Comp. Neurol. 361, 461–478. 10.1002/cne.903610310 8550893

[B16] Fahrenkrug J. Buhl T. Hannibal J. (1995). PreproPACAP-derived peptides occur in VIP-producing tumours and co-exist with VIP. Regul. Pept. 58, 89–98. 8577931 10.1016/0167-0115(95)00052-d

[B17] Galindo-Romero C. Jimenez-Lopez M. Garcia-Ayuso D. Salinas-Navarro M. Nadal-Nicolas F. M. Agudo-Barriuso M.. (2013). Number and spatial distribution of intrinsically photosensitive retinal ganglion cells in the adult albino rat. Exp. Eye Res. 108, 84–93. 10.1016/j.exer.2012.12.010 23295345

[B18] Glosmann M. Steiner M. Peichl L. Ahnelt P. K. (2008). Cone photoreceptors and potential UV vision in a subterranean insectivore, the European mole. J. Vis. 8, 23. 1–12. 10.1167/8.4.23 18484862

[B19] Greferath U. Grunert U. Wassle H. (1990). Rod bipolar cells in the mammalian retina show protein kinase C-like immunoreactivity. J. Comp. Neurol. 301, 433–442. 10.1002/cne.903010308 2262600

[B20] Guillery R. W. (2002). On counting and counting errors. J. Comp. Neurol. 447, 1–7. 10.1002/cne.10221 11967890

[B21] Hannibal J. Fahrenkrug J. (2004). Target areas innervated by PACAP immunoreactive retinal ganglion cells. Cell Tissue Res. 316, 99–113. 10.1007/s00441-004-0858-x 14991397

[B22] Hannibal J. Hindersson P. Knudsen S. M. Georg B. Fahrenkrug J. (2002a). The photopigment melanopsin is exclusively present in PACAP containing retinal ganglion cells of the retinohypothalamic tract. J. Neurosci. 22:RC191. 11756521 10.1523/JNEUROSCI.22-01-j0002.2002 PMC6757615

[B23] Hannibal J. Hindersson P. Nevo E. Fahrenkrug J. (2002b). The circadian photopigment melanopsin is expressed in the blind subterranean mole rat, Spalax. Neuroreport 13, 1411–1414. 10.1097/00001756-200208070-00013 12167764

[B24] Hannibal J. Kankipati L. Strang C. E. Peterson B. B. Dacey D. Gamlin P. D. (2014). Central projections of intrinsically photosensitive retinal ganglion cells in the macaque monkey. J. Comp. Neurol. 522, 2231–2248. 10.1002/cne.23555 24752373 PMC3996456

[B25] Hattar S. Kumar M. Park A. Tong P. Tung J. Yau K. W.. (2006). Central projections of melanopsin-expressing retinal ganglion cells in the mouse. J. Comp. Neurol. 497, 326–349. 10.1002/cne.20970 16736474 PMC2885916

[B26] Hattar S. Liao H. W. Takao M. Berson D. M. Yau K. W. (2002). Melanopsin-containing retinal ganglion cells: architecture, projections, and intrinsic photosensitivity. Science 295, 1065–1070. 10.1126/science.1069609 11834834 PMC2885915

[B27] Hattar S. Lucas R. J. Mrosovsky N. Thompson S. Douglas R. H. Hankins M. W.. (2003). Melanopsin and rod-cone photoreceptive systems account for all major accessory visual functions in mice. Nature 424, 76–81. 10.1038/nature01761 12808468 PMC2885907

[B28] Jagannath A. Hughes S. Abdelgany A. Pothecary C. A. Di Pretoro S. Pires S. S.. (2015). Isoforms of melanopsin mediate different behavioral responses to light. Curr. Biol. 25, 2430–2434. 10.1016/j.cub.2015.07.071 26320947 PMC4580334

[B29] Jain V. Ravindran E. Dhingra N. K. (2012). Differential expression of Brn3 transcription factors in intrinsically photosensitive retinal ganglion cells in mouse. J. Comp. Neurol. 520, 742–755. 10.1002/cne.22765 21935940

[B30] Janssen J. W. Bovee-Geurts P. H. Peeters Z. P. Bowmaker J. K. Cooper H. M. David-Gray Z. K.. (2000). A fully functional rod visual pigment in a blind mammal. A case for adaptive functional reorganization? J. Biol. Chem. 275, 38674–38679. 10.1074/jbc.M008254200 10984500

[B31] Janssen J. W. David-Gray Z. K. Bovee-Geurts P. H. Nevo E. Foster R. G. DeGrip W. J. (2003). A green cone-like pigment in the ‘blind’ mole-rat Spalax ehrenbergi: functional expression and photochemical characterization. Photochem. Photobiol. Sci. 2, 1287–1291. 10.1039/B300059C 14717222

[B32] Jusuf P. R. Lee S. C. Hannibal J. Grunert U. (2007). Characterization and synaptic connectivity of melanopsin-containing ganglion cells in the primate retina. Eur. J. Neurosci. 26, 2906–2921. 10.1111/j.1460-9568.2007.05924.x 18001286

[B33] La Morgia C. Ross-Cisneros F. N. Koronyo Y. Hnnibal J. Gallassi R. Cantalupo G.. (2015). Melanopsin retinal ganglion cell loss in Alzheimer's disease. Ann. Neurol. 79, 90–109. 10.1002/ana.24548 26505992 PMC4737313

[B34] Langel J. L. Smale L. Esquiva G. Hannibal J. (2015). Central melanopsin projections in the diurnal rodent, Arvicanthis niloticus. Front. Neuroanat. 9:93. 10.3389/fnana.2015.00093 26236201 PMC4500959

[B35] Lee E. S. Lee J. Y. Jeon C. J. (2010). Types and density of calretinin-containing retinal ganglion cells in mouse. Neurosci. Res. 66, 141–150. 10.1016/j.neures.2009.10.008 19895859

[B36] Lee S. C. Weltzien F. Madigan M. C. Martin P. R. Grunert U. (2016). Identification of A amacrine, displaced amacrine, and bistratified ganglion cell types in human retina with antibodies against calretinin. J. Comp. Neurol. 524, 39–53. 10.1002/cne.23821 26053777

[B37] Lucas R. J. Hattar S. Takao M. Berson D. M. Foster R. G. Yau K. W. (2003). Diminished pupillary light reflex at high irradiances in melanopsin-knockout mice. Science 299, 245–247. 10.1126/science.1077293 12522249

[B38] Milam A. H. Rose L. Cideciyan A. V. Barakat M. R. Tang W. X. Gupta N.. (2002). The nuclear receptor NR2E3 plays a role in human retinal photoreceptor differentiation and degeneration. Proc. Natl. Acad. Sci. U.S.A. 99, 473–478. 10.1073/pnas.022533099 11773633 PMC117584

[B39] Nadal-Nicolas F. M. Jimenez-Lopez M. Salinas-Navarro M. Sobrado-Calvo P. Alburquerque-Bejar J. J. Vidal-Sanz M.. (2012). Whole number, distribution and co-expression of brn3 transcription factors in retinal ganglion cells of adult albino and pigmented rats. PLoS ONE 7:e49830. 10.1371/journal.pone.0049830 23166779 PMC3500320

[B40] Nadal-Nicolas F. M. Salinas-Navarro M. Jimenez-Lopez M. Sobrado-Calvo P. Villegas-Perez M. P. Vidal-Sanz M.. (2014). Displaced retinal ganglion cells in albino and pigmented rats. Front. Neuroanat. 8:99. 10.3389/fnana.2014.00099 25339868 PMC4186482

[B41] Nemec P. Cvekova P. Benada O. Wielkopolska E. Olkowicz S. Turlejski K.. (2008). The visual system in subterranean African mole-rats (Rodentia, Bathyergidae): retina, subcortical visual nuclei and primary visual cortex. Brain Res. Bull. 75, 356–364. 10.1016/j.brainresbull.2007.10.055 18331898

[B42] Nevo E. Ivaniskaya I. Beiles A. (2001). Adaptive Radiation of Blind Subterrancan Mole Rats: Naming and Revisiting the Four Siblings Species of the Spalax Ehrenbergi Superspecies in Israel: Spalax Gallili (2n = 52), S. Golani (2n = 54), S. Carmeli (2n = 58) and S. Judaei (2n = 60). Leiden, Netherlands: Bachkhuys Publisher.

[B43] Ostergaard J. Hannibal J. Fahrenkrug J. (2007). Synaptic contact between melanopsin-containing retinal ganglion cells and rod bipolar cells. Invest. Ophthalmol. Vis. Sci. 48, 3812–3820. 10.1167/iovs.06-1322 17652756

[B44] Peichl L. Nemec P. Burda H. (2004). Unusual cone and rod properties in subterranean African mole-rats (Rodentia, Bathyergidae). Eur. J. Neurosci. 19, 1545–1558. 10.1111/j.1460-9568.2004.03263.x 15066151

[B45] Reifler A. N. Chervenak A. P. Dolikian M. E. Benenati B. A. Meyers B. S. Demertzis Z. D.. (2015). The rat retina has five types of ganglion-cell photoreceptors. Exp. Eye Res. 130, 17–28. 10.1016/j.exer.2014.11.010 25450063 PMC4276437

[B46] Rodriguez A. R. de Sevilla Muller L. P. Brecha N. C. (2014). The RNA binding protein RBPMS is a selective marker of ganglion cells in the mammalian retina. J. Comp. Neurol. 522, 1411–1443. 10.1002/cne.23521 24318667 PMC3959221

[B47] Sakai T. Calderone J. B. Lewis G. P. Linberg K. A. Fisher S. K. Jacobs G. H. (2003). Cone photoreceptor recovery after experimental detachment and reattachment: an immunocytochemical, morphological, and electrophysiological study. Invest. Ophthalmol. Vis. Sci. 44, 416–425. 10.1167/iovs.02-0633 12506104

[B48] Sanyal S. Jansen H. G. De Grip W. J. Nevo E. de Jong W. W. (1990). The eye of the blind mole rat, Spalax ehrenbergi. Rudiment with hidden function? Invest. Ophthalmol. Vis. Sci. 31, 1398–1404. 2142147

[B49] Schmidt T. M. Chen S. K. Hattar S. (2011a). Intrinsically photosensitive retinal ganglion cells: many subtypes, diverse functions. Trends Neurosci. 34, 572–580. 10.1016/j.tins.2011.07.001 21816493 PMC3200463

[B50] Schmidt T. M. Do M. T. Dacey D. Lucas R. Hattar S. Matynia A. (2011b). Melanopsin-positive intrinsically photosensitive retinal ganglion cells: from form to function. J. Neurosci. 31, 16094–16101. 10.1523/JNEUROSCI.4132-11.2011 22072661 PMC3267581

[B51] Schmidt T. M. Kofuji P. (2009). Functional and morphological differences among intrinsically photosensitive retinal ganglion cells. J. Neurosci. 29, 476–482. 10.1523/JNEUROSCI.4117-08.2009 19144848 PMC2752349

[B52] Schmidt T. M. Kofuji P. (2010). Differential cone pathway influence on intrinsically photosensitive retinal ganglion cell subtypes. J. Neurosci. 30, 16262–16271. 10.1523/JNEUROSCI.3656-10.2010 21123572 PMC3073605

[B53] Schmitz F. Konigstorfer A. Sudhof T. C. (2000). RIBEYE, a component of synaptic ribbons: a protein's journey through evolution provides insight into synaptic ribbon function. Neuron 28, 857–872. 10.1016/S0896-6273(00)00159-8 11163272

[B54] Sterling P. Matthews G. (2005). Structure and function of ribbon synapses. Trends Neurosci. 28, 20–29. 10.1016/j.tins.2004.11.009 15626493

[B55] Storchi R. Milosavljevic N. Eleftheriou C. G. Martial F. P. Orlowska-Feuer P. Bedford R. A.. (2015). Melanopsin-driven increases in maintained activity enhance thalamic visual response reliability across a simulated dawn. Proc. Natl. Acad. Sci. U.S.A. 112, E5734–E5743. 10.1073/pnas.1505274112 26438865 PMC4620906

[B56] Valiente-Soriano F. J. Garcia-Ayuso D. Ortin-Martinez A. Jimenez-Lopez M. Galindo-Romero C. Villegas-Perez M. P.. (2014). Distribution of melanopsin positive neurons in pigmented and albino mice: evidence for melanopsin interneurons in the mouse retina. Front. Neuroanat. 8:131. 10.3389/fnana.2014.00131 25477787 PMC4238377

[B57] Walmsley L. Hanna L. Mouland J. Martial F. West A. Smedley A. R.. (2015). Colour as a signal for entraining the Mammalian circadian clock. PLoS Biol. 13:e1002127. 10.1371/journal.pbio.1002127 25884537 PMC4401556

